# Viewing of Sexually Explicit Internet Material Among Jewish and Arab Adolescents: The Role of Parental Monitoring and Family Communication About Sexuality

**DOI:** 10.1007/s10508-025-03291-3

**Published:** 2025-12-19

**Authors:** Suha Daw, Alex Kharmatz, Miri Scharf

**Affiliations:** Department of Counseling and Human Development, University of Haifa, Abba Khoushy Ave 199, 3498838 Haifa, Israel

**Keywords:** Sexually explicit internet materials, Parental monitoring, Family communication regarding sexuality, Ethnocultural differences

## Abstract

The relation between familial characteristics and adolescent viewing of sexually explicit internet materials (SEIM) has been extensively studied. However, the impact of ethnocultural context on these relations remains largely unexplored. The present study aimed to address this gap by examining the moderating role of ethnocultural context in the associations between parental monitoring, family communication about sexuality, and viewing of SEIM. A total of 855 secular Jewish and Arab adolescents in Israel (58.5% females, *Mage* = 14.98 years, *SD* = 1.63) completed self-report questionnaires assessing these characteristics. Results showed that Jewish adolescents reported higher levels of family communication regarding sexuality and viewing of SEIM than Arab adolescents. While in both ethnocultural groups parental monitoring was negatively associated with viewing of SEIM, family communication regarding sexuality was negatively associated only with viewing of SEIM among Jews. In conclusion, our findings suggested that parental monitoring emerged as a potential protective factor against the viewing of SEIM. This factor may be more universal than culturally based. However, the effectiveness of family communication regarding sexuality in reducing the viewing of SEIM might be limited in Arab families. These findings emphasized the importance of taking the cultural context into account.

## Introduction

Adolescence is a developmental period during which sexual drives emerge, sexual behavior is initiated, and sexual attitudes and values are formed (Steinberg, 2020; Tolman & McClelland, 2011). Adolescents' understanding of their sexuality is shaped by their environment, while they face the challenge of navigating societal expectations, norms, and messages regarding sexuality (Goswam, 2023; Temple-Smith et al., 2015). Research shows that adolescents' sexual experiences and interactions with societal expectations and norms can impact their sexual self-concept, which may have long-term effects on their relationships and adult sexual functioning (Endendijk et al., 2022; Morgan, 2011).

The Internet has become one of the most common sources of sexual information and experiences for many adolescents today (Doornwaard et al., 2017; Kvardova et al., 2021). By browsing and sharing sexual materials, as well as engaging in virtual interactions, adolescents utilize the Internet to explore and express their sexual interests (Owens et al., 2012; Willis et al., 2022). However, concerns have been raised regarding the potential negative consequences of adolescents' viewing sexually explicit internet materials (SEIM) (Doornwaard et al., 2015). SEIM refers to online visual content that is sexually explicit, and viewed for the purpose of sexual arousal (McKee et al., 2020; Peter & Valkenburg, 2016). These materials often present an unrealistic portrayal of sexuality, characterized by excitement, casualness, and absence of risk, and frequently depict women as objectified and subordinate to sexually motivated men (Peter & Valkenburg, 2016; Ward, 2003). Adolescents may be susceptible, as their natural curiosity and limited real-life sexual experiences can make it critical assessment of the implications of the behaviors depicted in these materials challenging (Diamond & Savin-Williams, 2009). Consequently, without comprehensive sexual education and opportunities to foster the development of critical thinking skills, adolescents, whose cognitive capacities are still maturing, might be more likely to internalize and emulate the unrealistic portrayals of sexuality they encounter (Braun-Courville & Rojas, 2009; Ward, 2003). Frequent viewing of SEIM among adolescents was associated with the endorsement of permissive and recreational attitudes toward sexual activity (Owens et al., 2012; To et al., 2015), a higher risk of sexual violence and sexual addiction, and lower levels of satisfaction with sexual relationships (Morgan, 2011; O'Hara et al., 2013).

Both familial characteristics and broader cultural contexts influence the frequency of viewing SEIM (Kvardova et al., 2021; Peter & Valkenburg, 2016). According to Bronfenbrenner's (1979) ecological systems theory, adolescent behavior is shaped by interactions across various environmental levels, from immediate family dynamics (microsystem) to wider sociocultural values and norms (macrosystem). At the microsystem level, familial characteristics such as parental monitoring and family communication regarding sexuality (Kvardova et al., 2021; Peter & Valkenburg, 2016), play a key role in shaping adolescents’ sexual attitudes and behaviors (de Graaf et al., 2012; Van de Bongardt et al., 2018). However, the influence of these familial processes does not occur in isolation. Cultural norms and values within the macrosystem can either reinforce or reduce the effects of family-based protective factors (Hofstede, 2011; Sharabi, 2014).

Limited research has examined how the associations between family characteristics and adolescents’ viewing of SEIM may vary across different ethnocultural groups, particularly in multiethnic societies such as Israel. Given the distinct cultural approaches to sexuality observed among Jewish and Arab adolescents in Israel (Jabareen & Zlotnick, 2023; Khoury-Kassabri et al., 2005), this study explored whether ethnocultural affiliation moderated the associations between parental monitoring, family communication, and adolescents’ viewing of SEIM.

### Family Characteristics and Adolescents' Viewing of Sexually Explicit Internet Materials

According to the social learning theory (Bandura, 1986), adolescents learn behaviors by observing the actions and expectations of significant others in their environment, particularly parents. Prior research demonstrated that active parental monitoring, including awareness regarding adolescents’ daily activities and social networks, and encouraging them to share their experiences, was consistently associated with healthier sexual development and reduced risky behaviors among adolescents (de Graaf et al., 2012; Van de Bongardt et al., 2018). Furthermore, this was associated with reduced viewing of SEIM (Confalonieri et al., 2020; Khurana et al., 2019; Shanti & Gryselda, 2021; Tomić et al., 2018).

Moreover, open communication within families regarding sexuality facilitated the expression of thoughts and feelings, and helped adolescents manage various sexual situations (Laili et al., 2018; Rogers et al., 2022). Such communication may encourage critical thinking about sexual media messages (Koletić et al., 2019), and reduce reliance on sexual internet materials for sexual information (Sun et al., 2016; Warren, 2011). However, mixed findings indicated that family discussions on sexuality may lower barriers to viewing of SEIM (Doornwaard et al., 2015) and even predict increased viewing (Jaccard et al., 1996; Ward & Wyatt, 1994). Some studies found no association between family communication regarding sexuality and adolescents' viewing of SEIM (Peter & Valkenburg, 2006; Wolak et al., 2007). This lack of association might result from ineffective communication due to embarrassment, or denial of adolescent sexual activity rooted in cultural taboos (Somers & Paulson, 2000; Tesso et al., 2012).

In this study, parental monitoring refers to parental knowledge and adolescent disclosure (Stattin & Kerr, 2000), emphasizing adolescents’ active role in sharing information. Its relevance to SEIM and potential variation across cultural contexts remain underexplored. Furthermore, because communication about sexuality is shaped by cultural norms, it is considered especially pertinent for examining potential moderation by ethnocultural affiliation.

### The Moderating Role of Ethnocultural Affiliation

Hofstede’s (2011) cultural dimensions model offers a widely used framework for analyzing cross-cultural differences. Two dimensions—individualism versus collectivism and uncertainty avoidance—are particularly relevant for understanding how cultural context shapes family characteristics and adolescent behavior (Hofstede et al., 2010). In more individualistic cultures, which prioritize personal freedom and self-expression, parents often perceive their children as independent individuals capable of making their own decisions (Shuster et al., 2012). This leads to a more open family dialogue about sexuality, and a flexible approach to parental monitoring, emphasizing sharing and openness to help adolescents make informed choices (Kapetanovic et al., 2020; Lee et al., 2015). These cultures also exhibit low uncertainty avoidance, indicating high tolerance for ambiguity and flexibility (Hofstede et al., 2010; Smetana, 2017).

Conversely, more collectivist cultures emphasize group harmony and family reputation, characterized by high uncertainty avoidance and a preference for clear rules to minimize ambiguity (Hofstede et al., 2010). Within such cultures, parents tend to rely more on behavioral control strategies such as setting clear expectations, enforcing rules, and supervising observable behaviors, rather than fostering open, bidirectional dialogue with their children (Claes et al., 2018; Kocayörük et al., 2023). At the same time, adolescents in these cultural settings might feel less comfortable disclosing personal or potentially norm-violating behaviors due to concerns about shame or social repercussions (Gesser-Edelsburg & Abed Elhadi, 2018). Thus, limited adolescent openness might reduce the effectiveness of monitoring, and cultural norms may influence the dynamics that shape how monitoring unfolds rather than the willingness of parents to monitor. Similarly, family communication about sexuality may be less protective where it often lacks openness (Putnick et al., 2012; Withers et al., 2018), thereby reducing its potential to prevent adolescents' viewing of SEIM or to encourage critical thinking about such content.

Previous cross-cultural studies primarily focused on differences in adolescents’ overall levels of viewing SEIM across national or cultural contexts, generally showing higher viewing rates in Western countries compared to non-Western, more conservative cultures (e.g., Bleakley et al., 2009; Peter & Valkenburg, 2006; Ward, 2003). For example, Hald and Mulya (2013) found that adolescents in Western European countries reported significantly higher levels of viewing SEIM than their counterparts in Eastern European countries. These differences were often attributed to broader sociocultural norms regarding sexuality, with adolescents from individualistic cultures reporting more liberal sexual attitudes and greater openness toward SEIM, while adolescents from collectivistic cultures tended to report lower viewing levels and feelings of guilt following viewing (Coyne et al., 2019; Gesser-Edelsburg & Abed Elhadi, 2018). Further, Šmahel et al. (2014) found that adolescents from more conservative cultural backgrounds reported greater emotional distress and guilt following viewing of SEIM, highlighting how cultural values shape behavioral outcomes, as well as adolescents’ emotional interpretation of such experiences.

The few cross-cultural studies that considered family influences (e.g., Claes et al., 2018; Kocayörük et al., 2023), focused mostly on broader parenting styles rather than on these specific microsystem-level family processes in relation to viewing of SEIM. Given that proximal family processes may function differently under varying cultural norms (Bronfenbrenner, 1979; Hofstede, 2011), the present study extended existing literature by examining both the direct associations between family characteristics and adolescents’ viewing of SEIM, and whether these associations were moderated by ethnocultural affiliation.

To address this, we focused on adolescents living in Israel, a multiethnic society comprising Jews (74% of the population) and Arabs (21%) (Central Bureau of Statistics, 2023). The current study was conducted in the northern region of Israel, a socioculturally diverse area that includes both Jewish and Arab communities, and is home to the largest concentration of Arab populations in the country—including Muslims, Christians, and Druze (Central Bureau of Statistics, 2023). Participants were recruited from public (non-religious) Hebrew and Arabic language schools. In line with prior research (e.g., Khoury-Kassabri et al., 2005; Vitman-Schorr & Ayalon, 2020), the term “Arab” is used here to describe citizens of Israel who identify as members of the Arab minority, and who belong to one of the above religious communities.

The Jewish population in Israel is diverse in terms of religiosity and cultural orientation. There have been substantial social changes over the past decades in secular Jewish society, which comprises the majority of Jews in Israel, especially regarding gender roles and adolescent sexuality. These changes are reflected in more egalitarian family dynamics, lower levels of uncertainty avoidance, and greater openness toward adolescent sexual exploration (Mandel & Birgier, 2016; Sharabi, 2014). In contrast, Arab society in Israel is often described as a "society in transition" (Sabbah-Karkaby & Stier, 2017; Vitman-Schorr & Ayalon, 2020). While modernization processes have led to increased educational and occupational opportunities, traditional collectivist and patriarchal norms remain highly influential (Abu-Rabia-Queder, 2017). There is a strong cultural emphasis on family honor, modesty, and social conformity (Khoury-Kassabri et al., 2005) common to all three Arab religious groups. These groups also tended to exhibit higher collectivism and uncertainty avoidance, leading to more restrictive norms regarding adolescent sexual experiences, and less open communication about sexual matters (Abboud et al., 2015; El Kazdouh et al., 2019). In addition, sexual behavior during adolescence was widely stigmatized, with parental concerns often focusing on the adolescent's reputation and the family’s social standing (Dolev-Cohen & Ricon, 2022; Gesser-Edelsburg & Abed Elhadi, 2018). These shared cultural constraints were evident despite notable differences between the three Arab religious communities.

Despite residing in the same country and being subject to some influence from national laws, the cultural differences between the two ethnic groups (secular Jewish and Arabs) were nevertheless quite notable (Jabareen & Zlotnick, 2023; Pinquart & Silbereisen, 2005). Recent reports indicated that 80% of Jewish adolescents in Israel were exposed to SEIM, aligning with findings from Western countries, while 44% of Arab adolescents in Israel were exposed to such materials, consistent with non-Western and collectivist cultures (Efrati, 2023; Israel Internet Association, 2021; Jabareen & Zlotnick, 2023).

Given the cultural sensitivity surrounding adolescent sexuality in the Arab society, and the broader divergence in cultural norms between Jewish and Arab communities in Israel, this research offered valuable cross-cultural insights into how microsystem family characteristics may function differently under varying macrosystemic cultural influences concerning adolescents’ viewing of SEIM. Moreover, Arab adolescents are increasingly exposed to the norms, values, and media of the dominant Jewish majority culture, which may raise important questions about how competing cultural messages may shape adolescents' behaviors and family interactions.

### The Present Study

Building on Bronfenbrenner’s (1979) ecological systems theory, the present study examined the associations between familial characteristics, specifically parental monitoring (parental knowledge and adolescents' disclosure) and family communication regarding sexuality, and adolescents' viewing of SEIM. Additionally, we utilized Hofstede’s cultural dimensions framework (Hofstede, 2011), which provides a basis for interpreting ethnocultural differences between Jewish and Arab adolescents in Israel, to conceptualize these macrosystem-level cultural differences. The first aim of the study was to examine the associations between familial characteristics and viewing of SEIM among Jewish and Arab adolescents in Israel. We hypothesized that low levels of parental monitoring and family communication regarding sexuality would be associated with increased viewing of SEIM. The second aim was to examine ethnocultural differences in viewing of SEIM. We hypothesized that Jewish participants would report higher levels of viewing SEIM than Arab participants. Our third aim was to examine the potential moderation effect of ethnocultural affiliation on the associations between parental monitoring and family communication regarding sexuality, and adolescents' viewing of SEIM. Consequently, we hypothesized that the association between parental monitoring and family communication regarding sexuality would be stronger among Jewish adolescents than these associations among Arab adolescents.

To ensure the robustness of our findings, several demographic and individual characteristics associated with the frequency of viewing of sexual material were controlled. These included gender and grade level, as previous research shows that boys and older adolescents tend to view more SEIM than girls and younger adolescents (Nieh et al., 2020; Peter & Valkenburg, 2016). Similarly, we controlled for socioeconomic status and degree of religiosity, which have been found to negatively correlate with frequent viewing (Foubert & Rizzo, 2013; Higgins et al., 2022). Likewise, we controlled for sensation seeking, a personality trait often linked to increased viewing of SEIM (Khurana et al., 2019; Pikó & Pinczés, 2019). Additionally, we considered the frequency of sex education classes, as these programs are associated with lower viewing levels (Vandenbosch & Van Oosten, 2017). Since the study was conducted during COVID-19, we also controlled for changes in adolescents' viewing of SEIM habits during the pandemic, a period marked by increased screen time and altered viewing patterns of SEIM due to lockdowns (Maes & Vandenbosch, 2022). Lastly, given the expected significant differences based on gender and grade level, we examined the moderation of these variables on the associations between parental monitoring, family communication regarding sexuality, and viewing of SEIM.

## Method

### Participants

Participants were 855 adolescents (58.5% females) with a mean age of 14.98 (*SD* = 1.63), in eighth grade (60.9%) and eleventh grade (39.1%) at 17 public and secular secondary schools in northern Israel. In the sample, 45.6% of the participants were Jewish, and 54.4% were Arabs of various religions, including 26.2% Muslims, 24.3% Christians, and 3.9% Druze. Most of the participants' parents were well-educated (76.6% with higher education), and 85.5% of participants came from two-parent homes. Over two thirds (69%) of participants reported a moderate family income, and 26.5% a high family income. A chi-square test was conducted to examine differences between Arab and Jewish participants in socioeconomic status and parents' education. The findings indicated differences between Arab and Jewish participants in the family's economic status (${\upchi }^{2}\left(2\right)=6.36, p=.042)$; parents' marital status ${(\upchi }^{2}\left(2\right)=17.84, p<.001$); mother's education ${(\upchi }^{2}\left(3\right)=26.39, p<.001$), and father's education ${(\upchi }^{2}\left(3\right)=25.92, p<.001)$. Compared with Arab participants, Jewish participants reported higher economic status, higher level of academic education for both parents, and higher prevalence of divorced parents. These findings reflect the differences between Arabs and Jews in Israeli society, as described by the Central Bureau of Statistics (2023).

### Procedure

Adolescent participants and their parents gave active consent prior to participation. After receipt of approval from the schools, homeroom teachers distributed a virtual information sheet to parents, including a link to an online consent form. Only students whose parents provided active informed consent, and who themselves assented, were included in the sample. Data was collected in a group setting at school, during a 40-min period. The data collection was supervised by the principal researcher or research assistants. Respondents were asked to complete an online questionnaire on the Qualtrics website, using their mobile phones. Given the high prevalence of smartphone ownership among Israeli adolescents across ethnocultural groups, completing the survey via personal mobile phones during school hours was both feasible and suitable in all participating schools. Data collection took place from December 2021 to October 2022, with some participants (10.64% of the sample) completing the survey remotely due to COVID-19 pandemic restrictions. All the questionnaires were translated to Hebrew and Arabic by three independent Arabic-English speakers, and three independent Hebrew-English speakers. Final versions were created based on discussions and consensus on disagreements. In addition, a pretest of the version used was conducted with 30 eighth graders from each ethnocultural group.

## Measures

### Parental Monitoring

Selected subscales from the Perceived Parental Monitoring scale (PPM; Stattin & Kerr, 2000) were employed to assess parental knowledge and adolescent information sharing with parents. The "Parental Knowledge" subscale, consisting of 9 items, assesses the extent to which parents are informed about their children's activities (e.g., “Do your parents know what you do during your free time?”; Cronbach’s alpha = .86). "Adolescent Disclosure" subscale, consisting of 5 items, evaluates the extent to which adolescents voluntarily share their experiences with their parents (e.g., “If you are out at night, when you get home, do you tell your parents what you did that evening?”; Cronbach’s alpha = .78). All items were rated on 5-point Likert scale, ranging from “never” to “always”, with higher scores indicating greater levels of parental monitoring. Given the high correlation between the two subscales (*r* = .70, *p* < .001), we averaged all items to create a single composite parental monitoring score. Cronbach’s alpha for the combined scale was .89.

### Family Communication Regarding Sexuality

From the Family Sex Communication Quotient (Warren & Neer, 1986), eight items were chosen to examine two dimensions of family communication about sexuality: openness and informational content. Five items assess the perceived openness of communication with parents regarding sexuality-related topics (e.g., “I feel free to ask my parents questions about sexuality”), and three items assess the perceived amount of information shared during discussions on sexuality (e.g., “My parents have given me very little information about sexuality”). The term "parents" refers to both parents collectively, as clarified in the questionnaire instructions. A 5-point Likert scale was used, ranging from "strongly disagree" to "strongly agree*.*" Higher scores represent more frequent and comfortable family communication about sexuality. We averaged the eight items to create a total family communication regarding sexuality score. Cronbach’s alpha for the combined scale was .90.

### Viewing of Sexually Explicit Materials

The Sexually Explicit Internet Material Scale (Peter & Valkenburg, 2006) was employed, comprising four items that were utilized to assess the frequency with which adolescents intentionally viewed various forms of sexually explicit content over the previous six months. These items specifically inquired about the intentional viewing of (1) pictures with clearly exposed genitals; (2) movies with clearly exposed genitals; (3) pictures of people having sex; (4) movies of people having sex. Adolescents were informed that the question referred to sexual, pornographic Internet content. Responses were provided on a 7-point Likert scale, ranging from "never" to "several times a day", with higher scores indicating more frequent viewing. An overall SEIM viewing score was calculated by averaging the four items. Cronbach’s alpha was .95.

### Control Variables

Several control variables were taken into account, including gender and grade level.

#### Socioeconomic Status (SES)

Participants' socioeconomic status was assessed using two indicators: family SES and the school cultivation index. Family SES was based on three components: (1) self-reported assessment of the family’s economic standing (three-point Likert scale: below average, average, above average); (2) father’s highest level of education; and (3) mother’s highest level of education. Parental education was reported on a four-category scale: elementary school, high school, post-secondary, or academic education. All three components were standardized and averaged to create a composite family SES score. The school cultivation index, provided by the Israeli Ministry of Education, is a composite measure based on four weighted factors: the education level of the most educated parent (40%), family income per capita (20%), peripherality of the place of residence (20%), and a combined index of immigration status and family origin (20%). The resulting index is divided into five equally sized categories (quintiles), ranging from Quintile 1 (lowest SES) to Quintile 5 (highest SES).

#### Religiosity

A three-item scale (Bearman & Brückner, 2001) was used, which included: (1) the importance of religion (1 = "not important at all" to 5 = "extremely important"), (2) frequency of prayer, and (3) frequency of attending a place of worship (synagogue/mosque/church). The latter two items were rated on a 5-point Likert scale ranging from "never" to "several times a day". Composite religiosity score was computed by averaging the score of the three items, with higher scores indicating higher religiosity. Cronbach’s alpha was .85.

#### Sensation Seeking

The 8-item Brief Sensation Seeking Scale (BSSS; Hoyle et al., 2002) was used (e.g., “I would like to have new and exciting experiences, even if they are illegal”). Items were rated on a 5-point Likert scale ranging from "strongly disagree" to "strongly agree". Items were averaged to create a total sensation seeking score, with higher scores indicate higher levels of sensation seeking. Cronbach’s alpha was .72.

#### Frequency of Sex Education Classes

Participants were asked to assess the extent to which they had attended such classes at school. The exact question was:

“How often did you participate in sex education classes at school?” The options provided were: (1) Never, (2) Rarely, (3) Sometimes, (4) Often. Higher scores reflected more frequent participation in sex education classes.

#### Changes in Viewing of Sexually Explicit Internet Materials During COVID-19

To account for potential pandemic-related changes in viewing of SEIM, participants were asked: “To what extent did your viewing of SEIM change during the COVID-19 pandemic?” Response options were: (1) I watched less during the pandemic; (2) There were no changes; (3) I watched more during the pandemic.

#### Ethnocultural Affiliation

Participants were categorized based on the language of instruction at their schools (Arabic or Hebrew), which generally corresponds to students’ ethnocultural affiliation in the Israeli educational system. Although some Arab students attend Hebrew-speaking schools, none were included in the current sample.

### Data Analysis

Descriptive statistics were produced via two-way ANOVA and Pearson correlations. Furthermore, in order to test the hypothesis that ethnocultural affiliation moderated the association between adolescent's familial characteristics and viewing of SEIM, a moderation analysis was conducted using PROCESS Model 1 (Hayes, 2018). PROCESS is a regression-based add-on macro for testing moderation (i.e., interaction) effects and is particularly well-suited when the focal predictors are continuous, as in the present study. Given that PROCESS is limited to probe only one interaction term per analysis, we controlled for the other interaction terms via the covariate dialogue box, to ensure that all interaction terms were analyzed simultaneously. Significant moderation effects were then probed using simple slope approach.

## Results

### Descriptive Statistics

Table 1 presents descriptive statistics and correlations between the study variables. As summarized in Table 1, the viewing of SEIM was negatively associated with family communication regarding sexuality, and parental monitoring. In addition, the viewing of SEIM differed as a function of ethnocultural affiliation (see Table 1). Lastly, the theoretical predictors displayed weak to moderate correlations with each other, which indicated that they were fairly distinct theoretical concepts. Skewness and kurtosis were used to test for normality. Skewness values ranged from − 0.89 to 1.52, and kurtosis values ranged from − 0.49 to 1.24. According to the guidelines suggested by Hair et al. (2010), data can be considered approximately normal when skewness falls within the range of − 2 to + 2, and kurtosis between − 7 and + 7. Based on these criteria, the assumption of normality does not appear to be violated.

**Table 1:** Correlations between main study variables

	Mean	SD	Range	Skewness (SE)	Kurtosis (SE)	1	2	3
1. Viewing of SEIM	2.03	1.52	1–7	1.52 (0.09)	1.24 (0.17)			
2. Family communication regarding sexuality	2.71	.97	1–5	0.31 (0.09)	− 0.49 (0.17)	− .08^*^		
3. Parental monitoring	4.08	.75	1–5	− 0.89 (0.09)	0.32 (0.17)	− .28^***^	.32^***^	
4. Ethnocultural affiliation	–	–	–	–	–	− .12^***^	− .16^***^	.05

Ethnocultural affiliation: 0 = Jew, 1 = Arab

*N* = 855; * *p* < .05; **p < .01; *** *p* < .001

Given the sensitivity of the research topic, particularly in relation to cultural norms and gender, Table 2 reports differences in the main study variables by ethnocultural affiliation and gender, in order to provide a clearer understanding of how these factors may shape adolescents’ experiences and behaviors.

**Table 2:** Ethnocultural affiliation differences in the main study variables

	Jewish Boys	Jewish Girls	Arab Boys	Arab Girls	Ethnocultural affiliation	Gender	Ethnocultural affiliation × Gender
	M (SD)	M (SD)	M (SD)	M (SD)			
Viewing of SEIM	3.22 (1.75)	1.47 (0.88)	2.63 (1.39)	1.39 (1.44)	*F* = 12.96	*F* = 253.24	*F* = 7.25
					*p* < .001	*p* < .001	*p* = .007
					ηp^2^ = .015	ηp^2^ = .235	ηp^2^ = .009
Family communication regarding sexuality	2.81 (0.88)	2.92 (1.02)	2.54 (0.85)	2.58 (1.00)	*F* = 19.64	*F* = 1.41	*F* = 0.26
					*p* < .001	*p* = .236	*p* = .610
					ηp^2^ = .023	ηp^2^ = .610	ηp^2^ = .000
Parental monitoring	3.84 (0.72)	4.14 (0.77)	3.86 (0.78)	4.25 (0.68)	*F* = 1.53	*F* = 43.62	*F* = 0.83
					*p* = .217	*p* < .001	*p* = .362
					ηp^2^ = .002	ηp^2^ = .050	ηp^2^ = .001

Results of two-way ANOVA indicated significant ethnocultural and gender effects, which were qualified by ethnocultural × gender interaction. Simple effects revealed that compared to Arab girls, Jewish girls did not significantly view more SEIM (*p* = .479). However, Jewish boys viewed significantly more SEIM than Arab boys (*p* < .001). Moreover, for family communication, only significant main effect for ethnocultural affiliation was found. Jewish students reported more family communication regarding sexuality. With regard to parental monitoring, a main effect for gender was found. Girls reported more parental monitoring than boys.

### Moderation Effect of Ethnocultural Affiliation

In order to test the hypothesis that ethnocultural affiliation moderated the association between familial factors—parental monitoring and family communication about sexuality—and the viewing of SEIM, we conducted a moderation analysis via PROCESS macro in SPSS (Hayes, 2018). In addition, the moderation analysis accounted for the potential effects of seven sociodemographic variables: gender, grade level, perceived socioeconomic status, school cultivation index, religiosity, sensation seeking, frequency of sex education classes, and changes in viewing of sexual material during COVID-19. Moreover, we examined the moderation effects of gender and grade-level. All predictors were standardized prior to analysis to ease coefficient interpretation.

The model was statistically significant, *F*(17, 754) = 21.53, *p* < .001, with R-square of .312. As presented in Table 3, parental monitoring negatively predicted viewing of SEIM (*β* = -.12, *p* < .001), suggesting that higher parental monitoring was associated with decreased viewing of SEIM. However, the results indicated that family communication about sexuality was not significantly associated with the viewing of SEIM (*β* = -.03, *p* = .384). No significant ethnocultural affiliation effect was found (*β* = -.05, *p* = .165).

**Table 3:** The association between parental monitoring, and family communication regarding sexuality and viewing of sexually explicit internet materials, controlling for sociodemographic variables

	*Beta*	*SE*	*t*	*p*	*95%CI*
Parental monitoring	− .12	.03	3.59	< .001	[− .19, − .06]
Family communication regarding sexuality	− .03	.03	0.87	.384	[− .09, .04]
Ethnocultural affiliation	− .05	.04	1.39	.165	[− .12, .02]
Gender (girls)	− .42	.03	13.83	< .001	[− .48, − .36]
Grade (11th grade)	.04	.03	1.33	.184	[− .02, .10]
Cultivation index	.00	.03	0.02	.987	[− .07, .07]
Perceived Socioeconomic status	.07	.04	1.62	.106	[− .02, .16]
Religiosity	− .10	.03	2.93	.004	[− .17, − .03]
Sensation seeking	.10	.03	3.16	.002	[.04, .16]
Frequency of sex education classes	.03	.03	0.87	.385	[− .03, .09]
Changes in viewing of SEIM during COVID	.13	.03	4.51	< .001	[.08, .19]
Parental monitoring × Ethnocultural Affiliation	− .02	.03	0.50	.619	[− .08, .05]
Family communication regarding sexuality × Ethnocultural affiliation	.08	.03	2.40	.017	[.01, .14]
Parental monitoring × Gender	.06	.03	1.91	.057	[.00, .13]
Family communication regarding sexuality × Gender	− .03	.03	1.00	.318	[− .10, .03]
Parental monitoring × Grade	− .04	.03	1.11	.269	[− .10, .03]
Family communication regarding sexuality × Grade	.04	.03	1.22	.221	[− .02, .10]
*Simple slopes of family communication*					
Jews	− .11	.05	2.25	.024	[− .20, − .01]
Arabs	.05	.04	1.07	.286	[− .04, .13]

**Fig. 1:**
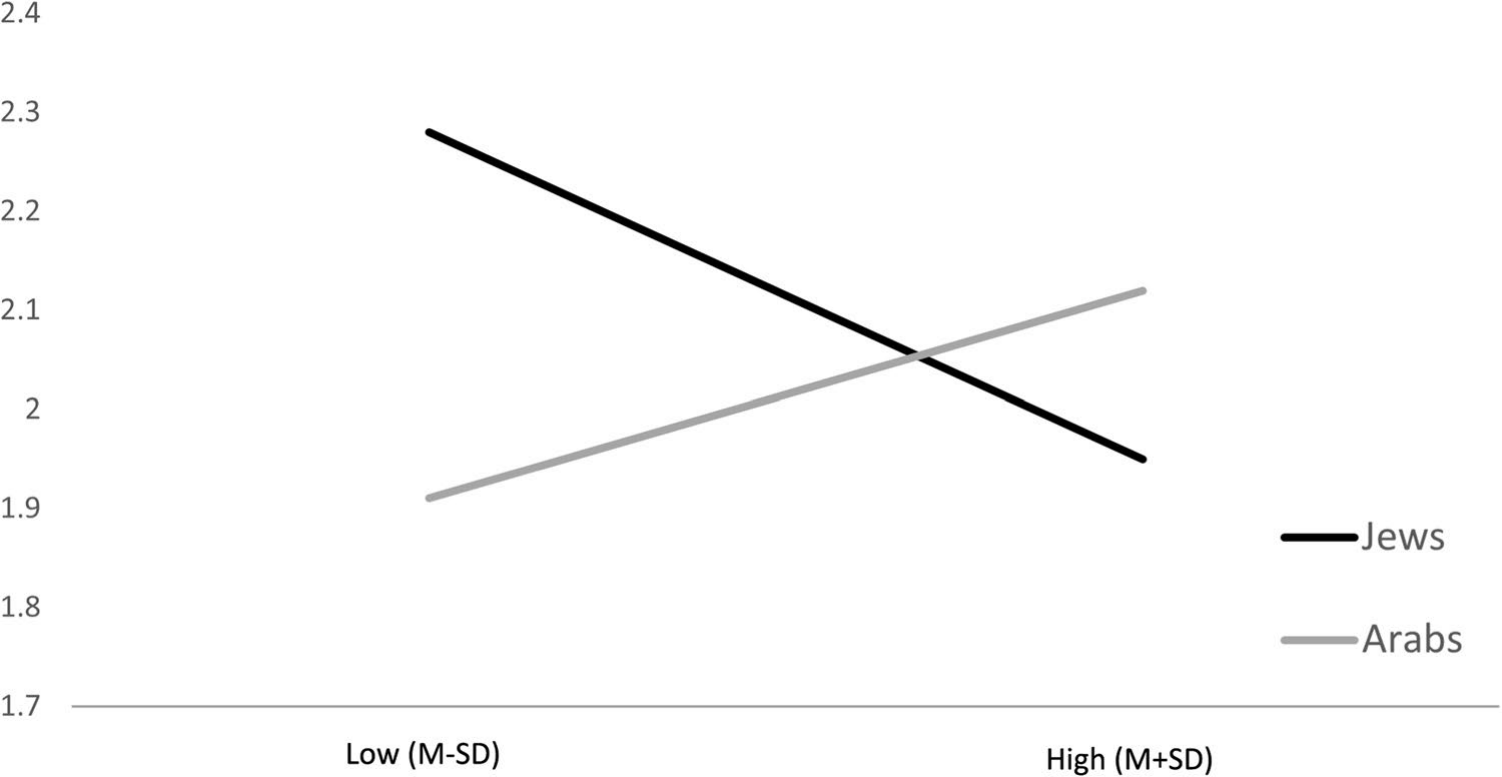
The conditional effects of family communication regarding sexuality on viewing of sexually explicit internet materials as a function of ethnocultural affiliation

Over and above the main effects, the results indicated that ethnocultural affiliation significantly moderated the association between family communication regarding sexuality and viewing of SEIM (*β* = .08, *p* = .017). Probing the interaction into conditional effects, as presented in the lower section of Table 3 and in Fig. 1, indicated that higher family communication regarding sexuality was related to decreased viewing of SEIM (*β* = -.11, *p* = .024) among Jews. Among Arabs, the association between family communication regarding sexuality and viewing of SEIM was not significant (*β* = .05, *p* = .286). It is also important to note that ethnocultural affiliation did not significantly moderate the association between parental monitoring and viewing of SEIM (*β* = -.02, *p* = .619).

Several sociodemographic variables were significantly associated with the viewing of SEIM. Results indicated a significant gender effect (*β* = -.42, *p* < .001), with boys (*M* = 2.92, *SD* = 1.82) viewing more SEIM than girls (*M* = 1.43, *SD* = .85). Higher levels of religiosity were associated with lower viewing of SEIM (*β* = -.10, *p* = .004). Sensation seeking was positively associated with viewing (*β* = .10, *p* = .002). Additionally, students who reported an increase in viewing during the COVID-19 pandemic indeed viewed more sexual material (*β* = .13, *p* < .001). In contrast, grade level, perceived socioeconomic status, frequency of sex education classes, and the school cultivation index were not significantly associated with viewing. Moreover, family communication about sexuality, and parental monitoring were not moderated by gender (*β* = .03, *p* = .318; *β* = .06, *p* = .057; respectively) nor by grade level (*β* = .04, *p* = .221; *β* = -.04, *p* = .269; respectively). Considered together, these findings indicated that the different association between family communication and viewing of SEIM in the two cultures was not attributed to sociodemographic variables. Moreover, neither gender nor grade-level attenuated the moderation effect of ethnocultural affiliation.

Given that Arab participants did not constitute a homogeneous group, we further examined potential differences across religious subgroups. Among the Arab participants, 47% (n = 213) identified as Muslim, 43% (n = 196) as Christian, 4% (n = 19) as Druze, and the remaining 6% (n = 27) either identified as others, or did not report their religious affiliation. Due to the small sample size of Druze participants, subsequent analyses focused solely on Muslims and Christians. Results indicated that Christian participants (*M* = 2.20, *SD* = 1.66) reported significantly higher viewing of SEIM than Muslim participants (*M* = 1.54, *SD* = 1.11), *t*(407) = 4.74, *p* < .001. In addition, Christians (*M* = 2.80, *SD* = 0.89) reported significantly more family communication about sexuality than Muslims (*M* = 2.40, *SD* = 0.93), *t*(407) = 4.39, *p* < .001. No significant differences emerged in parental monitoring between the two groups, *t*(407) = 0.35, *p* = .363.

Next, we examined whether religious subgroup membership moderated the associations between family communication and parental monitoring with viewing of SEIM, controlling for demography. Regression analyses revealed that religious subgroup did not significantly moderate the effect of family communication (*β* = .06, *p* = .526), or that of parental monitoring (*β* = –.13, *p* = .138). The absence of significant interaction effects suggested that the associations between family communication and parental monitoring with viewing of SEIM are consistent across Muslim and Christian participants. This finding further supported the notion that the key ethnocultural distinction lies between Arab and Jewish participants, rather than within subgroups of the Arab population.

## Discussion

### Ethnocultural Differences

Before delving into the main findings of the study, it is important to recognize the differences that we observed in the study variables based on adolescents' ethnocultural affiliation, as these provided a framework for the results of our study. Arab participants reported less family communication regarding sexuality than Jewish participants. These findings align with previous cross-cultural research, suggesting that cultural norms may influence families' willingness to discuss sexual topics with their adolescents (Gaither & Sellbom, 2003). In Arab culture, discouragement of norm-challenging behaviors and adolescents' sexual openness might contribute to limited family communication about sexuality (Abu-Baker, 2013; Dwairy & Achoui, 2010). In contrast, Jewish secular culture may be relatively more open and less conservative, allowing more family communication regarding sexuality (Assor et al., 2020). Interestingly, our findings revealed no ethnocultural differences in parental monitoring between Arab and Jewish families. This lack of difference may be due to common adolescent behaviors across cultures, where adolescents seek autonomy and privacy and tend to hide certain behaviors to avoid parental disapproval or punishment, and maintain family harmony (Smetana et al., 2009; Venkatraman et al., 2010; Yau et al., 2009).

As hypothesized, the findings indicated ethnocultural differences in the viewing of SEIM, with Arab adolescents reporting lower viewing than Jewish adolescents, in line with previous studies (Efrati, 2023; Israel Internet Association, 2021; Jabareen & Zlotnick, 2023). Social norms and boundaries that encourage modesty and sexual abstinence for Arab adolescents (Henry et al., 2007) may also result in their lower viewing of SEIM. These norms may stem from concerns about preserving family honor and adhering to conservative societal values (Khalaf & Gagnon, 2006).

Although the mean differences between Jewish and Arab adolescents in viewing of SEIM and parent–child communication were statistically significant, the effect sizes were small. These differences should not be over-interpreted at the individual level, but they may suggest broader, culturally influenced tendencies that could contribute to the development of culturally sensitive interventions or education programs. This pattern aligns with previous cross-cultural research, in which cultural effects on sexuality-related behaviors tended to be subtle, yet consistently observed.

### Association Between Parental Monitoring and Viewing of Sexually Explicit Internet Materials

The findings indicated that lower levels of parental monitoring and knowledge, and adolescents’ disclosure (components of parental monitoring) were associated with increased viewing of SEIM among adolescents. These findings aligned with Bronfenbrenner’s (1979) ecological systems theory, which emphasizes the role of proximal family processes, such as parental monitoring, in shaping adolescent behavior. Parental monitoring, as a component of the adolescent's microsystem, appears to function as a protective factor associated with lower viewing of SEIM. This is consistent with prior research that highlights the protective role of parental monitoring in mitigating risky behaviors, including the viewing of SEIM (Confalonieri et al., 2020; Fosco et al., 2012). Parental awareness of their children’s whereabouts and activities, which is facilitated by open disclosure from adolescents, plays a crucial role in creating a protective environment. This environment, characterized by trust and parental presence, can increase parental awareness of their children’s media viewing and, thereby, reduce their viewing of SEIM (Tomić et al., 2018; Weber et al., 2012; Zurcher, 2017). Moreover, adolescents who feel comfortable discussing their experiences with their parents may have a stronger sense of trust and openness in their relationship, might seek guidance from their parents and follow their advice, including avoiding SEIM (Boniel-Nissim et al., 2020; Olivari et al., 2018).

Our moderation analysis further explored whether ethnocultural affiliation moderated the association between parental monitoring and the viewing of SEIM. No moderating effect was found. This finding suggests that parental monitoring emerged as a potential protective factor against excessive viewing across different ethnocultural contexts.

### Association between Family Communication Regarding Sexuality and Viewing of Sexually Explicit Internet Materials

The findings underscored that the association between family communication regarding sexuality and the viewing of SEIM was influenced by cultural context. Among Jewish participants, open and comfortable communication about sexuality was associated with lower viewing of SEIM, supporting our hypothesis. Such communication may encourage adolescents to critically evaluate sexual media messages, and reduce their reliance on these materials (Sun et al., 2016; Wright, 2009). Additionally, open communication might assist parents in better understanding their children's needs, and provide guidance on managing viewing of SEIM, if necessary (Warren, 2011).

However, for Arab adolescents, the association between family communication regarding sexuality and the viewing of SEIM was not significant. This might suggest that (any) existing communication regarding sexuality in Arab families may not effectively reduce adolescents' viewing of SEIM. Possible explanations include a lack of parental awareness about their children's need for guidance on this topic, or challenges in discussing a taboo subject (Cok & Gray, 2007; Joubran, 2008). In these contexts, there might be a lack of proactive addressing of this issue during communication with their children, and difficulties discussing the topic openly. In addition, family communication may focus more on reinforcing societal norms and prohibitions than on fostering open discussions that promote critical thinking.

Despite controlling for several sociodemographic variables, the moderation effect of ethnocultural affiliation on the association between family communication regarding sexuality and viewing of SEIM remained significant. This finding aligned with Bronfenbrenner’s (1979) ecological systems theory, which emphasizes how macrosystem-level cultural values shape the functioning of microsystem-level family processes. Additionally, drawing on Hofstede’s cultural dimensions framework (Hofstede, 2011), Jewish adolescents—who affiliated with a more individualistic and lower uncertainty-avoidant culture—may experience family communication about sexuality as more open, supportive, and exploratory, making it an effective protective factor. In contrast, among Arab adolescents, who are socialized within a more collectivistic and high uncertainty-avoidant context, such communication might be more prescriptive and norm-reinforcing, limiting its protective role. These findings suggested that while open family communication may serve as a protective factor in more individualist cultures, it might be less effective in more collectivist cultures where societal norms play a stronger role in shaping behavior. This underscores the need for culturally tailored approaches when addressing adolescent viewing of SEIM.

### Limitations and Future Directions

The present study had several limitations. First, the study utilized a cross-sectional design, which limits the ability to draw causal conclusions. Future longitudinal studies could investigate the long-term effects of family interactions on adolescents' viewing of SEIM, and how changes in these factors during different stages of adolescence may affect viewing of SEIM. Second, considering that the study was conducted during the COVID-19 pandemic characterized by increased screen time, home confinement, and challenges in monitoring children's screen activities (Maes & Vandenbosch, 2022; Te Brinke et al., 2022), future research should investigate how family interactions, and ethnocultural affiliation influence adolescent viewing of SEIM under more routine conditions. Third, individualism and collectivism levels were not assessed at the individual level in the study. Considering this in future studies may minimize the risk of making broad generalizations about ethnocultural groups, and recognizing the diversity within each cultural background. Fourth, self-report questionnaires are vulnerable to social desirability biases, and this might be especially relevant when addressing sensitive topics such as parenting, sexuality and behaviors that might contradict social norms, particularly in conservative collectivist cultures (Durak & Seferoğlu, 2019; Rasmussen & Lauver, 2018). Future studies could employ multi-informant research by also obtaining parents’ perspective on parent-adolescent relationships. Future studies examining viewing of SEIM could utilize indirect questioning techniques, such as presenting hypothetical scenarios. For instance, participants could be asked to imagine discovering a website with explicit content, or receiving a sexual message on social media, and to express their level of interest or response to such situations. This approach may encourage less biased responses, as adolescents do not need to disclose their own actual viewing experiences. Finally, while the use of mobile phones for data collection might raise concerns regarding digital access, given the high prevalence of smartphone ownership among Israeli adolescents across both Jewish and Arab sectors (Israel Internet Association, 2021), this method was broadly accessible and unlikely to bias participation. Nevertheless, future research might consider collecting information on device ownership and digital access, particularly when studying online behaviors among adolescents. This would allow for a more nuanced understanding of how access to technology shapes both participation in research and viewing online content.

### Implications for Practice and Policy

The study findings carry several practical and policy-related implications. Given that parental monitoring was protective across both ethnocultural groups, interventions aimed at reducing adolescent viewing of SEIM should support parents in developing their ability to maintain awareness of their children's activities, and to foster adolescents' willingness to share information (Tomić et al., 2018; Zurcher, 2017). However, the protective effect of family communication about sexuality emerged only among Jewish adolescents. This highlights the need for culturally tailored communication-based interventions. In more conservative and collectivist contexts, such as the Arab society in Israel, programs should consider the cultural and social norms that inhibit open dialogue about sexuality (Gesser-Edelsburg & Abed Elhadi, 2018). Culturally sensitive school and community-based initiatives may help normalize these conversations in ways that respect familial and societal values (Sun et al., 2016). Policymakers are encouraged to develop sensitive, culturally responsive sex education curricula that reflect the differing needs of Arab and Jewish adolescents and their families. Overall, the study underscored the importance of culturally informed strategies that consider both family dynamics and broader societal norms when addressing adolescent viewing of SEIM.

### Conclusions

Viewing of SEIM during crucial stages of sexual identity development might be associated with adverse consequences (Huntington et al., 2024; Rousseau et al., 2021). Our findings highlighted the role of parental monitoring as a potentially universal protective factor across different cultural contexts. However, the effectiveness of family sexuality-related communication in reducing viewing of SEIM might differ across ethnocultural groups. For instance, in the less uniformly socialized Jewish community, open family discussions about sexuality could be associated with reduced viewing of sexually explicit materials, whereas this was not evident in the collectivist Arab community in Israel. Future studies could further examine these associations longitudinally throughout adolescence, as well as their associations with youth attitudes and behaviors. Understanding the nuanced role of ethnocultural context could help in developing tailored strategies to effectively address the viewing of SEIM and its potential consequences.

## Data Availability

The data and the custom code that support the findings of this study are available upon request from the corresponding author. Restrictions apply to the availability of these data due to the sensitive nature of the topic and the confidentiality of the participants.
